# Bringing dementia care to every corner of Tennessee: Implementing the Tennessee Dementia ECHO program

**DOI:** 10.1002/alz70858_104894

**Published:** 2025-12-26

**Authors:** Raymond R Romano, Rochelle Roberts, Sally Pitt, Daniel Ibarra‐Scurr, Anne Gifford, James Eaton, Katherine A. Gifford

**Affiliations:** ^1^ Vanderbilt Memory and Alzheimer's Center, Vanderbilt University Medical Center, Nashville, TN, USA; ^2^ Tennessee Department of Health, Nashville, TN, USA; ^3^ Vanderbilt Memory and Alzheimer's Center, Nashville, TN, USA; ^4^ Division of Geriatric Medicine, Vanderbilt University Medical Center, Nashville, TN, USA; ^5^ Department of Neurology, Vanderbilt University Medical Center, Nashville, TN, USA; ^6^ Framingham Heart Study, Boston University Chobanian & Avedisian School of Medicine, Boston, MA, USA

## Abstract

Like many other states in the United States, Tennessee is projected to see a 45% increase in residents over the age of 75 by 2040. Further, a projected 29‐fold increase in geriatricians will be required to meet care demands within the state. This impending situation will significantly limit access to timely dementia diagnoses and delay essential treatments, care planning, and support services. Research suggests investing in the broader primary care workforce, mainly through collaborative care models, can enhance health outcomes for individuals with dementia and reduce healthcare costs. The Tennessee Dementia ECHO program has been developed to address the critical need for a well‐prepared dementia care workforce in Tennessee. Launched in September 2024, the Tennessee Dementia ECHO program is a 12‐week tele‐mentoring initiative to improve primary care clinicians' and other healthcare professionals' knowledge, skills, and confidence in dementia care. Each one‐hour weekly session includes an expert‐led didactic presentation and an interactive case‐based discussion. Topics covered include dementia diagnosis, cognitive screening, medication management, addressing behaviors, and coordinating interdisciplinary care. The program attracted 63 participants from diverse healthcare roles, fostering a collaborative learning environment and building a network of dementia care providers across Tennessee. Participants were surveyed on their knowledge and confidence levels before and after each session, with results showing an average 50% increase in confidence, underscoring the program's effectiveness in empowering healthcare professionals. To aid rural healthcare providers, the ECHO program will create a registry of all providers who have completed the program and co‐design an electronic forum for continued education and collaboration. The Tennessee Dementia ECHO program demonstrates how targeted educational interventions can bridge workforce gaps, promote early detection and management of dementia, and improve health outcomes for older adults and their families. By continuing to expand and refine this initiative, Tennessee can serve as a model for other regions facing similar challenges.

